# Actuator-Assisted Calibration of Freehand 3D Ultrasound System

**DOI:** 10.1155/2018/9314626

**Published:** 2018-05-02

**Authors:** Terry K. Koo, Nathaniel Silvia

**Affiliations:** Foot Levelers Biomechanics Research Laboratory, New York Chiropractic College, Seneca Falls, NY, USA

## Abstract

Freehand three-dimensional (3D) ultrasound has been used independently of other technologies to analyze complex geometries or registered with other imaging modalities to aid surgical and radiotherapy planning. A fundamental requirement for all freehand 3D ultrasound systems is probe calibration. The purpose of this study was to develop an actuator-assisted approach to facilitate freehand 3D ultrasound calibration using point-based phantoms. We modified the mathematical formulation of the calibration problem to eliminate the need of imaging the point targets at different viewing angles and developed an actuator-assisted approach/setup to facilitate quick and consistent collection of point targets spanning the entire image field of view. The actuator-assisted approach was applied to a commonly used cross wire phantom as well as two custom-made point-based phantoms (original and modified), each containing 7 collinear point targets, and compared the results with the traditional freehand cross wire phantom calibration in terms of calibration reproducibility, point reconstruction precision, point reconstruction accuracy, distance reconstruction accuracy, and data acquisition time. Results demonstrated that the actuator-assisted single cross wire phantom calibration significantly improved the calibration reproducibility and offered similar point reconstruction precision, point reconstruction accuracy, distance reconstruction accuracy, and data acquisition time with respect to the freehand cross wire phantom calibration. On the other hand, the actuator-assisted modified “collinear point target” phantom calibration offered similar precision and accuracy when compared to the freehand cross wire phantom calibration, but it reduced the data acquisition time by 57%. It appears that both actuator-assisted cross wire phantom and modified collinear point target phantom calibration approaches are viable options for freehand 3D ultrasound calibration.

## 1. Introduction

**Figure 1:**
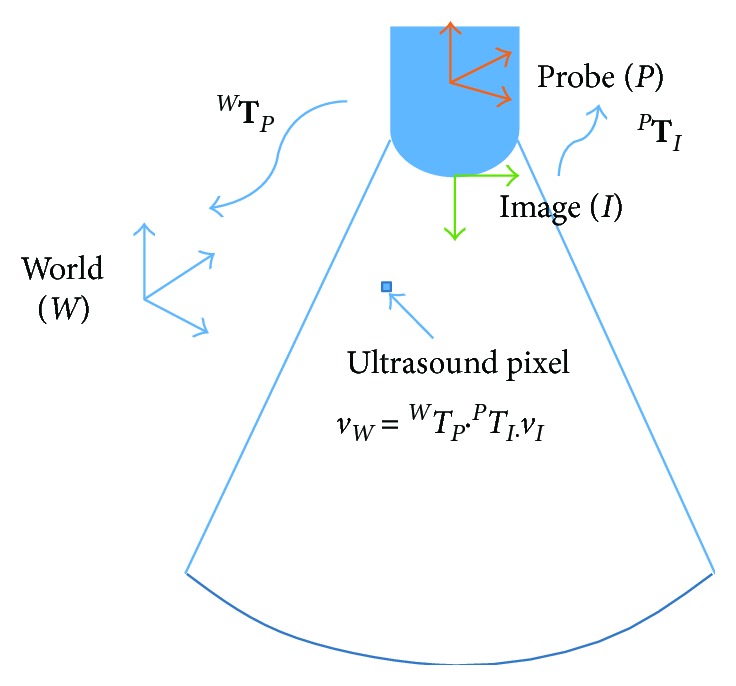
Principle of freehand 3D ultrasound. Each ultrasound pixel of an ultrasound image (*v*_*I*_) can be expressed with respect to the world coordinate system (*v*_*W*_) by multiplying two transformation matrixes (^*W*^**T**_*P*_ and ^*P*^**T**_*I*_): *v*_*W*_ = ^*W*^*T*_*P*_ · ^*P*^*T*_*I*_ · *v*_*I*_, where ^*P*^**T**_*I*_ and ^*W*^**T**_*P*_ are 4 × 4 transformation matrix relating the image coordinate system to the probe coordinate system and the probe coordinate system to the world coordinate system, respectively. Although ^*W*^**T**_*P*_ can be determined from the tracking system, ^*P*^**T**_*I*_ needs to be obtained through probe calibration.

Freehand three-dimensional (3D) ultrasound is a technique for acquiring 3D ultrasonic data of an anatomical feature of interest using a conventional two-dimensional (2D) ultrasound scanner. It has been used directly, or registered with other imaging modalities such as MRI and CT, to provide 3D visualization of the body for clinical volume measurement [1], analysis of complex geometries [2, 3], biomechanical analysis [4], surgical planning [5, 6], and radiotherapy planning [7, 8]. Briefly, freehand 3D ultrasound is accomplished by simultaneously acquiring 2D ultrasound images and tracking the position and orientation of an ultrasound probe using a mechanical, optical, or magnetic tracking system. Given that the tracking system can only record the position and orientation of the probe with respect to a world coordinate system (*W*), not the image plane with respect to (*W*), a probe calibration is required to obtain the rigid body transformation from the coordinate system of the ultrasound image to that of the probe (^*P*^**T**_*I*_) (Figure 1).

Probe calibration can be accomplished by scanning an object with known geometric dimensions called a phantom. The principle is to image the phantom, identify its features on the ultrasound images, and optimize the unknown transformation parameters that minimize the residual error between the sets of features identified in the images and on the phantom. Although several categories of calibration phantoms have been proposed (e.g., Z-fiducial [9, 10] and plane [11, 12] phantoms), point-based phantoms are still one of the most widely used [13–15], mainly because they are easy to build, accurate, and precise. However, probe calibration using point-based phantoms can be tedious and time-consuming. Point-based phantoms can either compose of single or multiple point targets made of bead, pin head, or intersecting wires. Among them, the single cross wire phantom has been commonly used as a reference standard to validate other calibration methods [12, 16], thanks to its excellent precision and accuracy. Traditionally, the single cross wire phantom requires an operator to repeatedly image the intersecting point in a freehand fashion [12, 17]. Transformation parameters from the coordinate system of the ultrasound image to that of the probe are then found iteratively without knowing the intersecting point locations [12, 18]. However, this approach requires the point targets to be scanned in multiple viewing angles [12]. Otherwise, the transformation parameters can be highly unconstrained by the optimization process and likely to be inaccurate [17]. It has also been suggested that a good cross wire phantom calibration requires the intersecting point to be imaged in all regions within the field of view (FOV) of the image plane [17]. Failure to do so may substantially deteriorate the precision and accuracy of the calibration. It is quite obvious that these “viewing angle” and “FOV” requirements make the traditional freehand cross wire phantom calibration highly skill dependent, and they may not be easily achieved in a timely manner.

The objective of this study was to refine the traditional freehand cross wire phantom calibration method by modifying the mathematical formulation of the calibration problem to eliminate the need of imaging the intersecting point at different viewing angles and developing an actuator-assisted approach to facilitate quick and consistent collection of intersecting points spanning the entire image FOV. We hypothesized that such refinements would enhance the precision and accuracy of the single cross wire phantom calibration and streamline its implementation. This hypothesis was tested by comparing the calibration reproducibility, point reconstruction precision, point reconstruction accuracy, distance reconstruction accuracy, and data acquisition time between the actuator-assisted and freehand cross wire phantom calibrations. To take advantage of the actuator-assisted calibration setup, we also constructed two phantoms with multiple point targets that could greatly simplify the alignment process of the point targets on the image plane, yet provide consistent collection of multiple target points that cover the entire image FOV in a timely manner. These new calibration approaches were also compared with the cross wire phantom calibrations in terms of precision, accuracy, and calibration time to evaluate their potential values for routine use.

## 2. Materials and Methods

### 2.1. Mathematical Formulation of the Calibration Problem

We measured the true coordinates of each point target *i* (where *i* = 1,2,3,…, *n*; *n* is the number of target points used for the calibration) with respect to the phantom coordinate system [10] using a digitizing probe (North Digital Inc.) with an accuracy of 0.1 mm in each direction and defined the true coordinates (${\bar{x}}_{{Ph}_{i}}\cdot y_{{Ph}_{i}}$, ${\bar{z}}_{{Ph}_{i}}$) as the average of 20 repeated measurements. Each point target *i* identified in the image coordinate system (*x*_*I*_*i*__, *y*_*I*_*i*__, 0) can be mapped to the phantom coordinate system (*x*_Ph_*i*__ · *y*_Ph_*i*__, *z*_Ph_*i*__) by

(1) xPhiyPhizPhi1=TPhWi·TWPi·TPI·sxxIisyyIi01,

where *s*_*x*_ and *s*_*y*_ are scaling factors in millimeters per pixel, which can be directly obtained by using the distance measurement tool provided by the ultrasound machine; (^Ph^**T**_*W*_)_*i*_ and (^*W*^**T**_*P*_)_*i*_ are 4 × 4 transformation matrix relating the world coordinate system to the phantom coordinate system and the probe coordinate system to the world coordinate system, respectively, and are given by the optical tracking system; and ^*P*^**T**_*I*_ is a transformation matrix relating the image coordinate system to probe coordinate system, which is an unknown calibration matrix governed by 6 independent parameters (3 rotations and 3 translations).

Hence, ^*P*^**T**_*I*_ can be found by minimizing the residual error (*D*) using nonlinear optimization among the *n* point targets 1, 2, 3,…, *n*:

(2) D=∑i=1nx¯Phiy¯Phiz¯Phi1−TPhWi·TWPi·TPI·sxxIisyyIi01n,

where |·| denotes the Euclidean distance between each corresponding point pair $\left[\left({\bar{x}}_{{Ph}_{i}}\cdot {\bar{y}}_{{Ph}_{i}},{\bar{z}}_{{Ph}_{i}}\right),\left(x_{{Ph}_{i}}\cdot y_{{Ph}_{i}},z_{{Ph}_{i}}\right)\right]$.

This mathematical formulation of the calibration problem allows for the calibration to be conducted with the probe positioned in one viewing angle only, which is a prerequisite of the actuator-assisted calibration as discussed in the next section.

### 2.2. Setup for Actuator-Assisted Calibration

**Figure 2:**
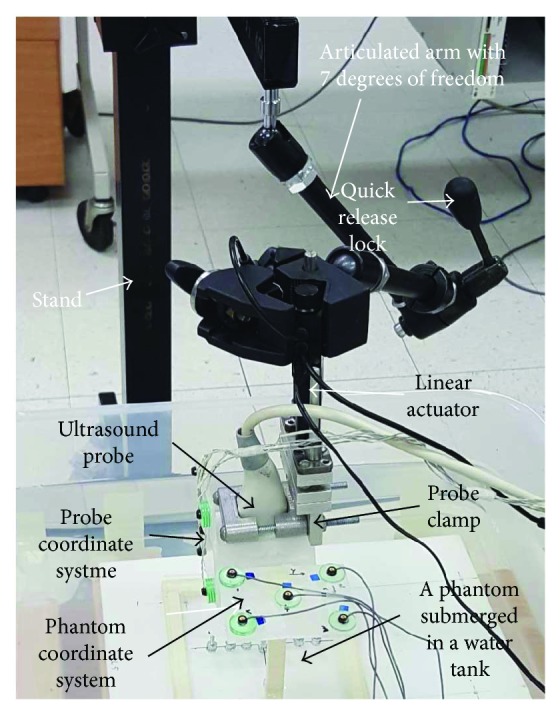
The setup for implementing the actuator-assisted calibration.

The purpose of actuator-assisted calibration was to facilitate imaging of multiple point targets over the entire image region in a systematic and time-efficient way. To accomplish this, a setup that consisted of a rigid stand, an articulated arm, a computer-controlled linear actuator, and a probe clamp was constructed to help position and hold the ultrasound probe perpendicular to point target(s) (Figure 2). Specifically, the articulated arm (Model 143, Manfrotto, Italy) possessed multiple degrees of freedom joints and a quick release lock to facilitate precise positioning and quick locking of the ultrasound probe. The articulated arm was rigidly connected to the stand at one end and the body of a linear actuator (T-NA08A50, Zaber Technologies, Canada) at the other end. A probe clamp was custom-made to allow for the probe to be mounted with its footprint perpendicular to the actuator shaft.

**Figure 3:**
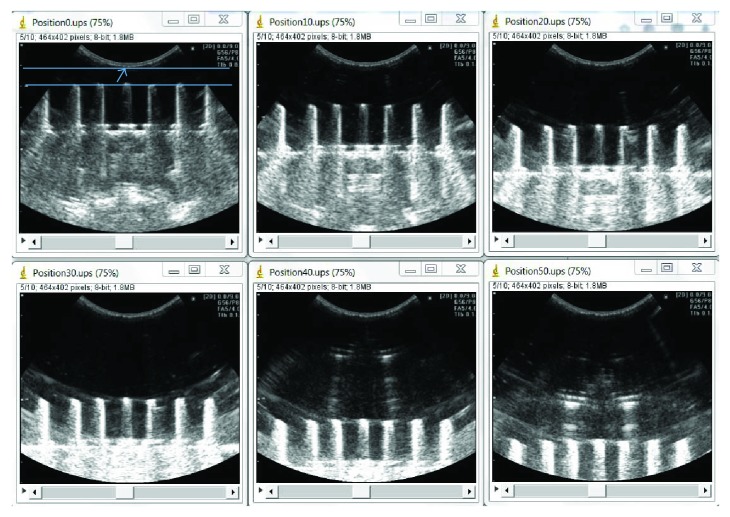
Ultrasound images of a collinear point target phantom after the one-time alignment process of the point targets. The top left image was captured with the linear actuator fully extended. The arrow indicates the lowest point of the footprint of the ultrasound probe, which is ~10 mm above the point targets (i.e., between the two parallel lines). The probe was retracted at 10 mm increment to cover the entire image field of view at 6 depth levels. Due to the curvilinear nature of the ultrasound probe used in this study, all of the 7 point targets can be clearly visualized at each depth level except for the most superficial level; only 5 screw heads can be visualized.

During actuator-assisted calibration, a phantom was first placed in a container that was filled with water at room temperature as a coupling media (Figure 2). With the shaft of the linear actuator at its fully extended position, the ultrasound probe was adjusted by unlocking the articular arm until (1) the lowest point of the footprint of the ultrasound probe was ~10 mm from the point target(s) of a phantom and (2) the image plane of the ultrasound probe is perpendicular to the point target(s) of a phantom (which was accomplished by aligning the spirit level on the probe clamp with the spirit level on the phantom base). Given that the linear actuator used in this study has a travel distance of 50 mm, the step (1) described above facilitated the point targets to be imaged over the entire FOV, whereas the step (2) allowed for point target(s) identified at one depth level to be imaged at other depth levels without the need of readjusting the probe orientation.  Figure 3 shows images of a phantom captured by the ultrasound probe following the alignment process. In most cases, the alignment process took less than a minute to accomplish.

### 2.3. Implementation of Actuator-Assisted Calibration

In this study, actuator-assisted calibration was implemented on three point-based phantoms: (1) single cross wire phantom, (2) original collinear point target phantom, and (3) modified collinear point target phantom. To facilitate a direct comparison of the precision and accuracy among phantoms, calibrations of all phantoms were based on 40 point targets using the same freehand 3D ultrasound system [4, 19] with depth and frequency settings of 90 mm and 4 MHz, respectively. The freehand 3D ultrasound system used in this study consisted of an ultrasound scanner (Ultramark 400c; ATL Ultrasound Inc., Bothell, WA) with a curvilinear probe (CLA 3.5/40), an optical tracking system (Optotrak 3020, Northern Digital Inc., Waterloo, Canada) with 5 noncoplanar infrared (IR) diodes attached on the ultrasound probe as well as on each phantom to keep track of the probe and phantom's pose with respect to the world coordinate system, and a personal computer with a frame grabber and a data acquisition card installed for capturing ultrasound images. Although 3 noncollinear diodes are sufficient to track the pose of a rigid body, the use of 5 IR diodes increases the accuracy of the pose estimation because of the inherent averaging of individual IR diode position errors when the corresponding poses are determined [20]. Given that the phantom coordinate system (Ph) of each phantom could be arbitrarily defined for the purpose of probe calibration, three of the 5 IR diodes were arbitrarily selected to establish a phantom coordinate system (Ph) for each phantom (Figure 2). First, an IR diode was selected as the origin. Second, we defined the *x*-axis as the unit vector from the origin to the 2nd IR diode. Third, a temporary axis was defined as the unit vector from the origin to the 3rd IR diode. Fourth, *z*-axis was defined as the cross product of the temporary axis and the *x*-axis. Finally, the *y*-axis was defined as the cross product of the *z*-axis and the *x*-axis. A probe coordinate system (*P*) was also established in a similar way by mounting 5 IR diodes on the lateral surface of the probe (Figure 2). Thanks to actuator-assisted calibration setup, both of the ultrasound probe and the phantom are stationary after alignment process, and hence ultrasound images and pose data can be acquired independently without the need of synchronization.

#### 2.3.1. Cross Wire Phantom

The cross wire phantom consisted of two coplanar nylon wires attached at the top of a plastic box (100 × 100 × 80 mm) with their intersection (i.e., the point target) located at the center of the box opening. During the actuator-assisted calibration, the probe was positioned at 6 different depth levels by the linear actuator, each separated by 10 mm. To ensure that the 40 point targets covered the entire image region, the intersecting point was imaged at 7 different locations along the lateral dimension of an image plane at each depth level, except for the most superficial level. Only 5 locations were imaged at this level due to its smaller lateral dimension.

To better understand the potential benefits of actuator-based calibration, the ultrasound probe was also calibrated with the same cross wire phantom but using a traditional freehand cross wire phantom calibration approach [12]. Given that the ultrasound probe cannot be regarded as stationary during freehand cross wire phantom calibration, images and pose data were synchronized by aligning a 5 V pulse that was sent to both of the ultrasound and Optotrak computers. In addition, actuator-assisted cross wire phantom calibration was conducted with all target points localized along the center region of the image FOV to explicitly evaluate the effects of point target distribution on calibration accuracy and precision. Again, 40 point targets were imaged for each trial to allow for a direct comparison of the precision and accuracy with the actuator-based calibration.

#### 2.3.2. Original Collinear Point Target Phantom

**Figure 4:**
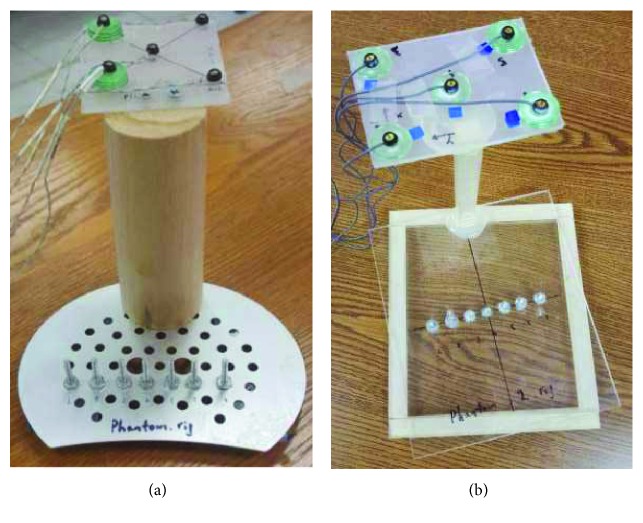
(a) The original collinear point target phantom; (b) the modified collinear point target phantom. The top plate of the modified collinear point target phantom is slightly rotated about the central screw to better illustrate the phantom design.

The original collinear point target phantom comprised 7 collinear screws (diameter: 2 mm, interscrew distance: ~15 mm) mounted perpendicular to an aluminum plate with the screw heads serving as point targets (Figure 4(a)). This arrangement eliminated the need to move the phantom laterally during a calibration, as the entire lateral dimension of the image plane was well covered by the point targets. The alignment process of the point targets was as follows: after the probe was properly positioned perpendicular to the phantom with the point targets at ~40 mm depth, the phantom was carefully adjusted until all 7 target points were clearly seen in the B-mode image. Ultrasound images and pose data of the phantom and probe were then acquired at 6 different depth levels, each separated by 10 mm. Altogether, 40 point targets were imaged in 6 images (7 point targets at each depth level except for the most superficial level, where only 5 point targets could be visualized) (Figure 3).

#### 2.3.3. Modified Collinear Point Target Phantom

The modified collinear point target phantom also consisted of 7 collinear screws. However, it was built differently to facilitate alignment of the image plane with the 7 point targets in a more systematic and time-efficient manner. It comprised a base plexiglass plate (152.4 mm × 177.8 mm) with a screw mounted at its center, a top plexiglass plate of the same dimension with a central hole of 2 mm diameter and 6 screws (3 on each side of the central hole), and they were stacked together as in Figure 4(b). This design allowed for the top plate to rotate about the central screw yet maintained the collinearity among the 7 screws, facilitating independent adjustment of the translational and rotational degrees of freedom during the alignment process of the point targets. We anticipated that this design would improve the precision and accuracy of the calibration. Like the original collinear point target phantom, we acquired ultrasound images and pose data at 6 depth levels.

### 2.4. Segmentation and Speed Correction

All point targets (*x*, *y*) were manually segmented using ImageJ (NIH, Bethesda) by a research assistant, who was blinded to the calibration results. The coordinates of each point target were also corrected for speed of sound distortion (*δx*, *δy*) based on the ray model proposed by Goldstein [21] such that the corrected coordinates of each point target became (*x* + *δx*, *y* − *δy*). Specifically, we measured water temperature after each calibration trial and plugged it into a fifth-order polynomial equation [22] to calculate the actual speed of sound (*c*_a_) for each calibration trial. Assuming the footprint of the curvilinear probe is a circular arc, we calculated its radius of curvature (*R*) and origin location. This allowed us to calculate the shift in lateral (*δx*) and axial (*δy*) directions as follows:

(3) $$\begin{array}{c} \delta x=\left(D_{im}-R\right)\left(1-\frac{c_{a}}{c_{cal}}\right)\sin\theta ,\\ \delta y=\left(D_{im}-R\right)\left(1-\frac{c_{a}}{c_{cal}}\right)\cos\theta , \end{array}$$

where *D*_im_ is the distance between the origin and the segmented point target within the image plane, *c*_cal_ = 1540 m/s is the assumed speed of sound used by the ultrasound machine, and *θ*  is the angle between a line from the origin to the segmented point target and a line along the axial direction. *θ*  is positive if the segmented point target is located at the left half of the image and vice versa.

### 2.5. Performance Evaluation

Altogether, 5 calibration approaches were compared in this study: (1) freehand cross wire phantom calibration, (2) actuator-assisted cross wire phantom calibration, (3) actuator-assisted cross wire phantom calibration based on point targets at the central region, (4) actuator-assisted calibration using an original collinear point target phantom, and (5) actuator-assisted calibration using a modified collinear point target phantom. Ten calibration trials were conducted for each calibration approach. Data acquisition time was recorded for each calibration trial. The probe alignment process was performed for each actuator-assisted calibration trial to allow for a more realistic evaluation of the actuator-assisted approaches.

For each calibration trial, we optimized 6 independent calibration parameters (*α*, *β*, *γ*, *x*, *y*, and *z*) of ^*P*^**T**_*I*_. Although the probe coordinate system could be arbitrarily defined for the purpose of probe calibration, we strategically attached the 5 IR diodes and defined the probe coordinate system such that its *x*-, *y*-, *z*-axis approximated the elevation, lateral, and axial directions of the ultrasound beam, respectively, to facilitate our interpretation of the sources of uncertainty for different calibration approaches. Hence, (*α*, *β*, and *γ*) could be interpreted as the Euler angles in *z*-*y*-*x* (or axial-lateral-elevation) sequence that specified the orientation of the image coordinate system with respect to the probe coordinate system, and (*x*, *y*, and *z*) could be interpreted as the translations along the elevation, lateral, and axial directions of the ultrasound beam, respectively, that located the origin of the image coordinate system with respect to the probe coordinate system. To this end, we reported the standard deviation of each calibration parameter among the 10 calibration trials for each calibration approach.

#### 2.5.1. Precision

Precision refers to how close measurements are to each other. In this study, we quantified precision using two widely used parameters: calibration reproducibility [12] and point reconstruction precision [12]. To quantify the calibration reproducibility, we transformed the 4 corners and the middle of the image [23] from the image to the probe space using the calibration parameters of each of the 10 calibration trials, calculated the Euclidean distance between all possible pairs of the 10 possible calibration trials (i.e., _10_*C*_2_ = 45 pairs) for each point, and reported the mean, standard deviation, maximum, and minimum of the pooled data of the 5 points (i.e., 225 observations). To calculate point reconstruction precision, we fixed a different point phantom to the world space. This point phantom consisted of a plastic base with a single pin, 2 mm in diameter, affixed to the base. We imaged the pin head 50 times at different viewing angles and locations within the entire image FOV and derived the world coordinates of the pin head from different views and calibration matrix combinations. From there, we further calculated the Euclidean distance between all possible pairs of views (i.e., _50_*C*_2_ = 1225 pairs) for each calibration parameter set and reported the mean, standard deviation, maximum, and minimum of the pooled data of the 10 calibrations (i.e., 12,250 observations). This was done for each of the 5 calibration approaches. Detailed mathematical formulations of both precision parameters can be found elsewhere [12].

#### 2.5.2. Accuracy

Accuracy refers to how close measurements are to the “true” value. For each calibration approach, we quantified reconstruction accuracy using both point-based and distance-based measures. For the point reconstruction accuracy [24], we compared the world coordinates of the pin head derived from each view and calibration matrix combination (obtained from the “point reconstruction precision” experiment described above) with the true world coordinates (measured by the digitizing probe based on the average of 20 measurements) and reported their differences in terms of mean, standard deviation, maximum, and minimum among 500 observations (i.e., 50 views × 10 calibration trials). An additional validation experiment was conducted using a “two-point” phantom (i.e., a plastic base with two pins, 2 mm in diameter, affixed to the base) to quantify distance reconstruction accuracy [10, 12, 18]. This involved (1) digitizing each point target 20 times to calculate the true 3D distance between the two point targets, (2) imaging each point target 50 times at random viewing angles and locations that covered the entire image FOV in a qualitative manner, (3) deriving the “imaged” 3D distance between the two point targets based on each image pair (50 × 50 = 2500 image pairs) and calibration matrix (10 calibration trials) combinations, and (4) calculating the difference between the true and imaged 3D distances in terms of mean, standard deviation, maximum, and minimum among 25,000 observations.

#### 2.5.3. Statistical Analysis

For each parameter (i.e., calibration reproducibility, point reconstruction precision, point reconstruction accuracy, distance reconstruction accuracy, and data acquisition time), one-way analysis of variance (ANOVA) was employed to test whether there was a significant difference among the 5 calibration approaches. Post hoc comparisons were based on the Scheffe's method. All statistical tests were done using SPSS statistical package version 24 (SPSS, Chicago, IL). A confidence level of 0.05 was chosen for all analyses.

## 3. Results

Residual error indicates the proximity of the locations of the 40 point targets used for each calibration with respect to their true locations after optimization. It provides an indication of self-consistency of the calibration. It was noted that the freehand cross wire phantom calibration resulted in the largest residual error (1.35 ± 0.21 mm), followed by the actuator-assisted cross wire phantom calibration (0.80 ± 0.11 mm), and the actuator-assisted cross wire phantom calibration based on point targets at the central region had the smallest residual error (0.40 ± 0.09 mm). The residual errors of both original (0.55 ± 0.04 mm) and modified (0.47 ± 0.08 mm) collinear point target phantom calibrations were also very small.

Tables 1 and 2 summarize the calibration reproducibility and point reconstruction precision, respectively, for each calibration approach. We found that the actuator-assisted cross wire phantom calibration was the most precise among all calibration approaches. However, if point targets were only imaged at the central region of the image FOV, its precision deteriorated tremendously. In addition, modified collinear point target phantom appears to be slightly better than the original collinear point target phantom in terms of both precision measures.

Tables 3 and 4 summarize the point and distance reconstruction accuracy of each calibration approach, respectively. As expected, the accuracy of the actuator-assisted cross wire phantom calibration was poor if the point targets were imaged at the central region of the image FOV only. All other calibration approaches appear to have excellent but similar accuracy.

**Figure 5:**
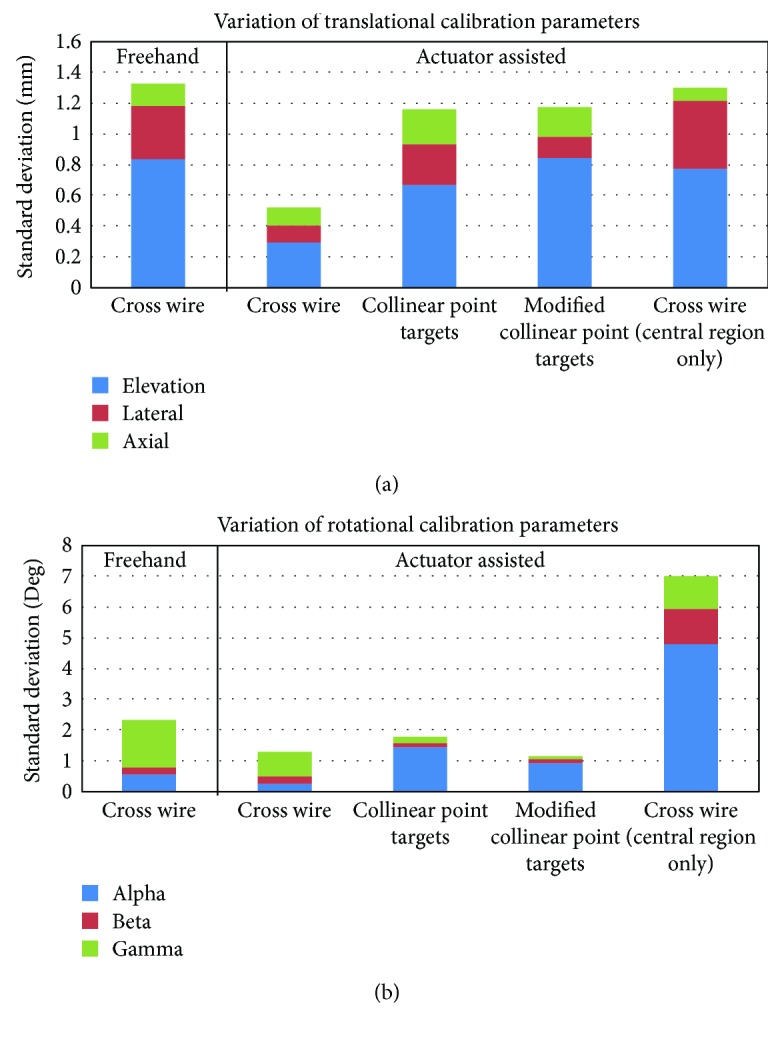
Variation of (a) translational and (b) rotational calibration parameters among the 5 calibration approaches evaluated in this study.

To help elucidate the sources of uncertainty for each calibration approach, standard deviations of the translational (*x*, *y*, and *z*) and rotational calibration parameters (*α*, *β*, and *γ*) for each calibration approach are also plotted as separate stacked columns (Figure 5).

Table 5 summarizes the data acquisition time for each calibration approach. Results revealed that actuator-assisted calibration was particularly time-efficient if it was implemented with collinear point target phantoms. However, similar data acquisition time was recorded for both freehand and actuator-assisted cross wire phantom calibration approaches.

## 4. Discussion

Our accuracy and precision data (Tables 1–4) compared favorably with other calibration methods reported in the literature [17, 25]. However, because of difference in the quality of ultrasound system, probe frequency and configuration, depth settings, number of target points, accuracy of the tracking system and segmentation, and so on, accuracy and precision data must be interpreted with extreme caution. We overcame this difficulty by using the traditional freehand cross wire phantom calibration as a reference standard in this study. Among the four calibration approaches tested in this study (i.e., freehand cross wire phantom calibration, actuator-assisted cross wire phantom calibration, actuator-assisted collinear point target phantom calibration, and actuator-assisted modified collinear point target phantom calibration), the actuator-assisted single cross wire phantom calibration significantly outperformed the other calibration approaches in terms of calibration reproducibility (Table 1) and appeared to perform slightly better in terms of point reconstruction precision and accuracy (Tables 2 and 3) but similar to the other calibration approaches in terms distance reconstruction accuracy (Table 4). Figure 5 further revealed that actuator-assisted single cross wire phantom calibration substantially improved the consistency of both translational and rotational calibration parameters, especially the translational parameters. It is worth noting that calibration reproducibility depends only on the calibration parameters, but point reconstruction precision, point reconstruction accuracy, and distance reconstruction accuracy depend on both the calibration parameters and errors associated with reconstruction (e.g., segmentation and tracking errors). That may explain why point reconstruction precision, point reconstruction accuracy, and distance reconstruction accuracy were less distinctive between calibration approaches. Nonetheless, one could comfortably conclude that the overall performance of the actuator-assisted cross wire phantom calibration is the best among all calibration approaches tested in this study.

It has been suggested that probe calibration using point-based phantom should ensure that the point targets be imaged in all regions within the FOV [17]. The results of this study not only confirmed this notion but also elucidated the reason behind that. We demonstrated that if the point targets were only imaged at the central region of the image FOV during cross wire phantom calibration, even though the residual error of the calibration was among the smallest (likely due to better image quality at the center of the FOV), the rotational calibration parameters (especially rotation about the axial direction) became highly unconstrained (Figure 5(b)), resulting in tremendous deterioration of precision (Tables 1 and 2) and accuracy (Tables 3 and 4).

We successfully modified the mathematical formulation of the calibration problem to eliminate the need of imaging the point targets at different viewing angles. Although this modification would benefit the traditional freehand cross wire phantom calibration, it offers additional benefits to the actuator-assisted cross wire phantom calibration. First, with the probe held by a probe clamp instead of by hand, the intersecting points can be more precisely located within the ultrasound midplane (reflected by a smaller variation of the translational calibration parameter along the elevation direction (Figure 5(a))). Second, given that both of the probe and the phantom are stationary during imaging, there is no need to synchronize the pose data with the ultrasound images. Third, with the ultrasound probe connected to a linear actuator in the actuator-based calibration, we can easily fulfill the FOV requirement by systematically adjusting the actuator's positions to cover the entire FOV of ultrasound image.

In this study, we developed 2 phantoms with collinear point targets to take advantage of the actuator-assisted calibration setup. Like the cross wire phantom, phantoms with collinear point targets can be easily constructed in a nonengineering setting. Our results revealed that their precision and accuracy were comparable to those of the traditional freehand cross wire phantom calibration yet significantly reduced the calibration time from 26.2 minutes to 11.1 minutes. Based on the validation data of the original collinear point target phantom, we had already identified that the rotational component about the axial direction and the translational component along the elevation direction were the main sources of uncertainty (Figure 5). In fact, these components are primarily governed by the alignment process of the point targets. Hence, we intended to develop the modified collinear point target phantom to improve the alignment process of the point targets. As expected, the new alignment mechanism improved the rotational precision about the axial direction (Figure 5(b)), but surprisingly, it degraded the translational precision along the elevation direction (Figure 5(a)). This is likely because the modified collinear point target phantom only relied on the central screw head to guide the translational alignment whereas the original collinear point target phantom used all the 7 screw heads. Nonetheless, side-by-side comparison of each precision (Tables 1 and 2) and accuracy (Tables 3 and 4) parameter as well as the data acquisition time revealed that the modified collinear point target phantom appears to be slightly better than the original collinear point target phantom.

Due to differences in phantom design, the implementation of the actuator-assisted calibration requires repeated alignment of each of the 40 point targets with the image plane for the single cross wire phantom, whereas the collinear point target phantoms only require one alignment for all the 40 point targets. In the first glance, one may think that collinear point target phantoms are more attractive choices for the implementation of actuator-assisted calibration. However, the “single” alignment approach of collinear point target phantoms could introduce a systematic error to all the 40 point targets. Given that the amplitude and direction of this systematic error may vary substantially among calibration trials (due to the subjective nature of visual alignment), its precision is likely to be compromised. Conversely, the “repeated” alignment requirement of the single cross wire phantom would lead to a random error to each point target, and hence the calibration should be less sensitive to point target alignment error. This may explain why the actuator-assisted cross wire phantom calibration was more precise than the actuator-assisted collinear point target phantom calibrations but at the same time, had a larger residual error. This observation also brought up the fact that residual error is simply an indication of self-consistency of a calibration. It may be useful for detecting a poor calibration trial by comparing the residual error with other calibration trials of the same calibration approach. However, it is not a measure of precision or accuracy, and hence it should not be directly compared between calibration approaches.

The actuator-assisted calibration approach developed in this study is not without limitation. First, due to the fact that our current setup is mainly composed of metal parts (e.g., linear actuator, probe clamp, stand, and articulated arm), the optical tracking system may not be replaced by magnetic tracking device. This makes our current setup less portable. Further development should focus on replacing the metal components by plastic components. Second, unlike the traditional approach, our approach requires the use of specific components such as digitizing pointer and linear actuator, which may not be commonly found in some scenarios, especially in clinical settings. Third, we only evaluated our actuator-assisted calibration approach on 3 point-based phantoms; its applicability to other point-based phantoms is largely unknown. It will be a topic of future study.

## 5. Conclusion

In conclusion, we successfully developed an actuator-assisted approach to make the freehand 3D ultrasound calibration less skill dependent, applied it to a single cross wire phantom and two collinear point targets phantoms, and evaluated their precision and accuracy by comparing with the traditional freehand cross wire phantom calibration approach. Results demonstrated that the actuator-assisted single cross wire phantom calibration significantly improved the calibration reproducibility and offered similar point reconstruction precision, point reconstruction accuracy, distance reconstruction accuracy, and data acquisition time with respect to the freehand cross wire phantom calibration. On the other hand, the actuator-assisted modified collinear point target phantom calibration was found to have similar precision and accuracy when compared to the freehand cross wire phantom calibration, but it reduced the data acquisition time by 57%. It appears that both actuator-assisted approaches are viable options for freehand 3D ultrasound calibration.

## Figures and Tables

**Table 1:** Comparison of calibration reproducibility among calibration approaches.

	Freehand cross wire	Actuator assisted cross wire	Actuator assisted collinear point targets	Actuator assisted modified collinear point targets	Acutator assisted cross wire (central region only)
Mean (SD)	1.50 (1.08)	0.84 (0.57)^∗^	1.72 (1.20)	1.50 (0.95)	4.91 (3.99)^#^
Maximum	5.29	2.85	5.10	4.99	15.94
Minimum	0.043	0.042	0.13	0.077	0.14

For each approach, calibration reproducibility was calculated from 225 observations. All data in mm. ^∗^One-way ANOVA revealed a significant difference among calibration approaches. Post hoc analysis further revealed that calibration reproducibility of the actuator-assisted cross wire phantom calibration was significantly better than that of the traditional freehand cross wire phantom calibration (*p* = 0.015). ^#^However, if actuator-assisted cross wire phantom calibration was only focused on the central region, it was significantly poorer than the traditional freehand cross wire phantom calibration (*p* < 0.0001). Other actuator-based calibration approaches were not significantly different from the traditional freehand cross wire phantom calibration.

**Table 2:** Comparison of point reconstruction precision among calibration approaches.

	Freehand cross wire	Actuator assisted cross wire	Actuator assisted collinear point targets	Actuator assisted modified collinear point targets	Acutator assisted cross wire (central region only)
Mean (SD)	1.69 (1.04)	1.44 (0.83)	1.68 (1.13)	1.49 (0.92)	2.86 (2.46)^#^
Maximum	6.03	4.63	7.85	5.91	17.38
Minimum	0.035	0.045	0.037	0.046	0.039

For each approach, point reconstruction precision was calculated from 12,250 observations. All data in mm. ^#^One-way ANOVA revealed a significant difference among calibration approaches. Post hoc analysis further revealed that if actuator-assisted cross wire phantom calibration was only focused on the central region, point reconstruction precision was significantly poorer than the traditional freehand cross wire phantom calibration (*p* = 0.009). Other actuator-based calibration approaches were not significantly different from the traditional freehand cross wire phantom calibration.

**Table 3:** Comparison of point reconstruction accuracy among calibration approaches.

	Freehand cross wire	Actuator assisted cross wire	Actuator assisted collinear point targets	Actuator assisted modified collinear point targets	Acutator assisted cross wire (central region only)
Mean (SD)	1.44 (0.82)	1.33 (0.74)	1.65 (0.80)	1.35 (0.75)	2.61 (1.59)^#^
Maximum	3.84	3.64	4.59	4.17	9.89
Minimum	0.14	0.11	0.21	0.12	0.32

For each approach, point reconstruction precision was calculated from 500 observations. All data in mm. ^#^One-way ANOVA revealed a significant difference among calibration approaches. Post hoc analysis further revealed that if actuator-assisted cross wire phantom calibration was only focused on the central region, point reconstruction accuracy was significantly poorer than the traditional freehand cross wire phantom calibration (*p* < 0.0001). Other actuator-based calibration approaches were not significantly different from the traditional freehand cross wire phantom calibration.

**Table 4:** Comparison of distance reconstruction accuracy among calibration approaches.

	Freehand cross wire	Actuator assisted cross wire	Actuator assisted collinear point targets	Actuator assisted modified collinear point targets	Acutator assisted cross wire (central region only)
Mean (SD)	0.19 (0.47)	0.19 (0.46)	0.21 (0.48)	0.21 (0.48)	0.244 (0.74)^#^
Maximum	1.88	1.79	1.90	1.82	4.70
Minimum	−1.62	−1.64	−1.61	−1.56	−2.37

For each approach, point reconstruction precision was calculated from 25,000 observations. All data in mm. ^#^One-way ANOVA revealed a significant difference among calibration approaches. Post hoc analysis further revealed that if actuator-assisted cross wire phantom calibration was only focused on the central region, distance reconstruction accuracy was significantly poorer than the traditional freehand cross wire phantom calibration (*p* = 0.024). Other actuator-based calibration approaches were not significantly different from the traditional freehand cross wire phantom calibration.

**Table 5:** Comparison of data acquisition time among calibration approaches.

	Freehand cross wire	Actuator assisted cross wire	Actuator assisted collinear point targets	Actuator assisted modified collinear point targets
Mean (SD) (minutes)	26.2 (5.1)	26.0 (4.0)	12.8 (3.4)^∗^	11.1 (3.7)^∗^

For each approach, mean (SD) was based on 10 calibration trials. ^∗^One-way ANOVA revealed a significant difference among calibration approaches. Post hoc analysis further revealed that data acquisition time for both actuator-based collinear point targets and modified collinear point target phantom calibrations was significantly shorter than that for the traditional freehand cross wire phantom calibration (*p* < 0.0001 for each paired comparison). There was no significant difference in data acquisition time between freehand and actuator-assisted cross wire phantom calibration.
